# Supplementary material for: The causal relationship between the human gut microbiota and pyogenic arthritis: a Mendelian randomization study
Source: Front Cell Infect Microbiol. 2024 Nov 26;14:1452480. doi: 10.3389/fcimb.2024.1452480 (PMC11629706; doi:10.3389/fcimb.2024.1452480)
Supplement: Supplementary file 1 [file DataSheet1.zip › mendelian test/ebi-a-GCST90027811.csv_forest.pdf]

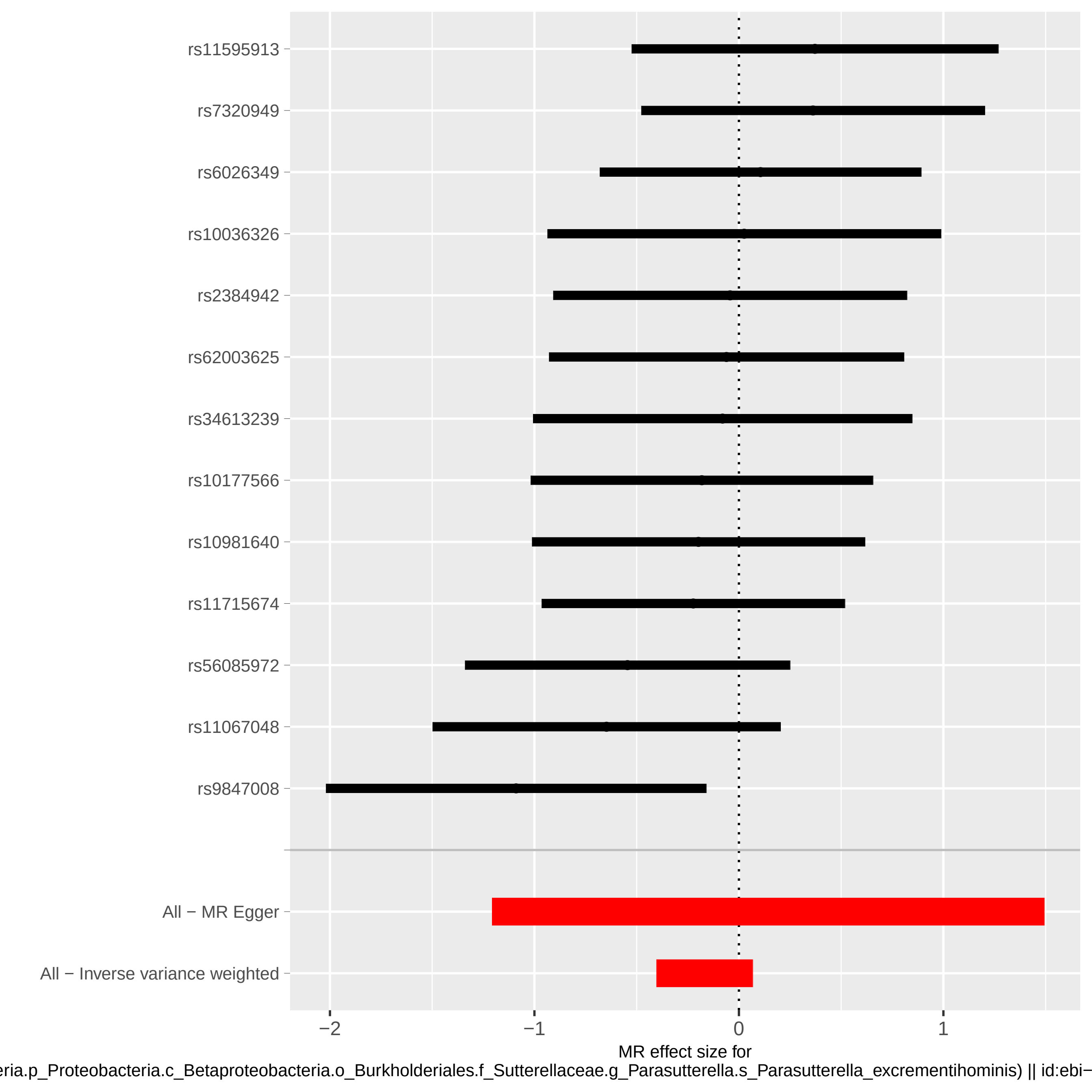
rs11595913
rs7320949
rs6026349
rs10036326
rs2384942
rs62003625
rs34613239
rs10177566
rs10981640
rs11715674
rs56085972
rs11067048
rs9847008
All − MR Egger
All − Inverse variance weighted
−2
−1
0
1
MR effect size for
eria.p_Proteobacteria.c_Betaproteobacteria.o_Burkholderiales.f_Sutterellaceae.g_Parasutterella.s_Parasutterella_excrementihominis) || id:ebi−
